# Isatuximab‐carfilzomib‐dexamethasone immediately after failing of the quadruplet Daratumumab‐bortezomib‐lenalidomide‐dexamethasone (Dara‐VRD): Striking response with no washout in a newly diagnosed multiple myeloma

**DOI:** 10.1002/ccr3.8449

**Published:** 2024-01-23

**Authors:** Juan José Gil‐Fernández, Patricia García Ramírez, Marta Callejas Charavía

**Affiliations:** ^1^ Hematology Department University Hospital Principe de Asturias Alcala de Henares Spain

**Keywords:** daratumumab‐refractory myeloma, first‐line therapy, isatuximab post daratumumab, newly diagnosed multiple myeloma

## Abstract

Biochemical evolution of serum IgG‐Kappa monoclonal component during the first line with VRD (x1), DARA‐VRD (x4), and the second line with ISA‐KD (x4).

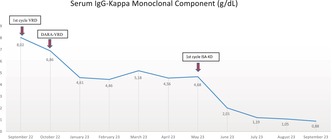

## 1 INTRODUCTION

Nowadays, in transplant‐eligible multiple myeloma (MM) patients, the combinations of three or four drugs commonly known as triplets or quadruplets (a proteasome inhibitor or PI, an immunomodulatory drug or IMiD, dexamethasone +/− an anti‐CD38 monoclonal antibody or mAb) have become a well‐established standard for induction therapy.^1^ These combinations obtain the best response rates (very good partial responses‐ VGPR‐ or better >70%–90%) after performing an autologous stem cell transplantation (ASCT).^1^, ^2^

However, what should be done if a quadruplet combination as daratumumab‐bortezomib‐lenalidomide‐dexamethasone (Dara‐VRD) produces a poor response (e.g., a minimal or partial response). In this scenario, the use of an isatuximab combination,^3^ immediately after a daratumumab‐containing regimen and with no washout period might be controversial. Many myeloma attending physicians might avoid the sequence of isatuximab after daratumumab because of the poor clinical results reported in the literature with a second CD38‐targeted mAb after failing to a first.^4^, ^5^

We are here presenting the fast, deep, and striking response achieved with isatuximab‐carfilzomib‐dexamethasone (ISA‐KD) in a patient with a minimal response after four cycles of Dara‐VRD. This isatuximab combination produced an excellent response that allow us to proceed to ASCT in a VGPR status.

## 2 CASE REPORT

In October 2022, while on holidays at the Canary Islands, a 54‐year‐old Caucasian female was diagnosed of a community‐acquired pneumococcal pneumonia that required hospitalization. At admission, she exhibited severe anemia, acute renal failure, and significant increase of serum proteins. The main abnormal laboratory parameters were as follows: hemoglobin 7.1 g/dL, creatinine 1.91 mg/dL, EGF 29 mL/min, serum total proteins 12.3 g/dL, serum albumin 2.5 g/dL, an IgG‐Kappa monoclonal component (MC) 8.02 g/dL in the serum protein electrophoresis, and B2‐microglobulin 8 mg/L with normal calcium and LDH. Whole body low‐dose CT scan showed no osteolytic bone lesions. Bone marrow studies (cytology, flow cytometry, and biopsy) revealed a diffuse generalized infiltration by medium‐sized and immature plasmacytic cells representing a 90% of the nucleated cells that expressed CD38 + low, CD138++, sIgKappa+, cyIgKappa+, CD28+, and Cyclin D1+, being negative for CD45, CD19, CD56, and CD117; FISH analysis showed a rearrangement of the genes CCND1‐MYEOV/IgH indicative of the presence of t(11;14) (q13.3;q32.3), with no deletion of 17p13, no deletion of 1p36/1p32, and no gain or amplification of 1q21. A symptomatic MM at stage ISS‐III, R‐ISS II, presenting with a severe infectious complication was diagnosed.

After antibiotic treatment, the patient was transferred to Madrid. She underwent induction chemotherapy with subcutaneous bortezomib 1.3 mg/m2 on days 1, 8, 15, and 22, lenalidomide 25 mg orally per day for 14 days and dexamethasone 40 mg orally on days 1, 8, 15, and 22 (VRD) for one cycle. On day +11, she developed a generalized and symptomatic cutaneous rash and cotrimoxazole, allopurinol, and lenalidomide were withdrawal. After consultation with the allergology department, a sulfonamides allergy was confirmed. Subcutaneous daratumumab 1800 mg on days 1 and 15 was added to the VRD (Dara‐VRD) and peripheral blood stem cells were harvested after the second cycle. After four cycles, the patient achieved only a minimal response (IgG‐Kappa MC 4.68 g/dL) and was readmitted due to a second community‐acquired pneumonia with *Haemophilus influenzae* bacteriemia.

**FIGURE 1:**
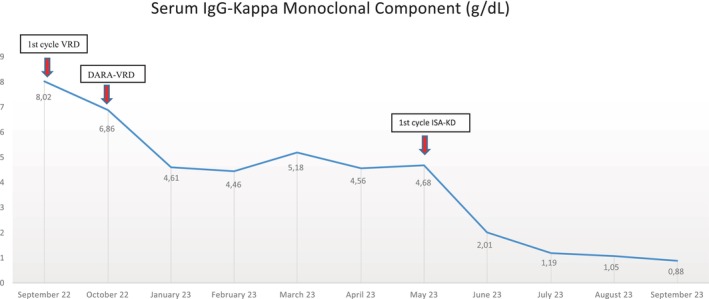
Biochemical evolution of serum IgG‐Kappa monoclonal component during the first line with VRD (x1), DARA‐VRD (x4), and the second line with ISA‐KD (x4).

Due to this poor response, without a washout period, we decided to administer a second‐line treatment with an approved and financed isatuximab combination. The period free of a CD38‐targeted mAb was of 35 days, between the last daratumumab dose on April 10th and the first isatuximab administration on May 15th. Isatuximab, carfilzomib, and dexamethasone (ISA‐KD) were planned, with a once‐weekly carfilzomib dose of 70 mg/m2 on days 1, 8, and 15 (A.R.R.O.W. scheme) every 4 weeks.^6^ Simultaneously, intravenous immunoglobulins at a dose of 0.4 g/Kg every 4 weeks were administered as anti‐infectious prophylaxis. After completing the first cycle of ISA‐KD (four doses of isatuximab), a faster and deeper response was observed compared to the previous four cycles of Dara‐VRD (reduction of MC from 4.68 g/dL to 2.01 g/dL). Following four cycles of ISA‐KD with a status of nearly VGPR (MC of 0.88 g/dL), the patient is scheduled to undergo an ASCT (see evolution of biochemical response at Figure 1).

## 3 DISCUSSION

Patients with newly diagnosed MM (NDMM) nonresponding to or progressing after an initial quadruplet induction therapy (e.g., Dara‐VRD) have a worse outcome compared to primary responders.^7^, ^8^ In the GRIFFIN study, the large majority of NDMM responded favorably to the Dara‐VRD four drugs combination (>VGPR 90% after consolidative ASCT) with only a 6.7% of disease progression under therapy.^1^ Effective rescue treatments for this population of poorly responding or primary refractory MM who failed to a quadruplet therapy are not well established.

The two CD38‐targeted mAb approved for use in MM are daratumumab and isatuximab. Both anti‐CD38 mAb have different mechanisms of action, targeting different epitopes of the CD38 molecule at several cell lines (MM, T, and NK cells) to produce their effects on malignant MM and immunologic microenvironment cells surrounding them. Furthermore, the mechanisms of drug resistance are also different for both anti‐CD38 mAb as has been extensively investigated.^9^, ^10^

In 2021, Mikhael J et al published the unfavorable outcomes with isatuximab monotherapy in 32 heavily pretreated Refractory Relapsing (RR) MM patients in a Phase 2 prospective multicenter study.^4^ These patients had received a median of seven prior lines, all had a daratumumab‐refractory MM and all were also refractory to their last therapy (progressive disease during treatment or within 60 days of treatment discontinuation). A 60% of patients received a daratumumab combination just prior to isatuximab and in 62.5% of patients the interval between last daratumumab dose and first isatuximab administration was less than 6 months. As expected, the clinical outcomes were poor with an overall disease control rate (defined as more than minimal response or stable disease >8 weeks) of 37.5%. Disease control seemed to improve with a longer interval between the last daratumumab dose and the first isatuximab, giving rise to the concept of “washout” period (time without receiving an anti‐CD38 mAb). Mikhael J et al observed that patients with a washout period exceeding 6 months achieved disease control in 58.3% of cases versus 28.6% for those with a washout period of less than 3 months. These poor results observed with isatuximab monotherapy after failing to daratumumab in this heavily pretreated and worse prognosis population, has had a big impact on the clinical practice, and many hematologists are now usually waiting a washout period of 3–6 months, before considering the use of isatuximab combinations in daratumumab‐refractory MM.^4^

Olivia Perez de Acha et al in “ex vivo” laboratory assays termed Myeloma Drug Sensitivity Testing (My‐DST) performed on 37 patients' bone marrow aspirates from 29 extensively pretreated and multirefractory MM patients demonstrated that MM cells from daratumumab‐exposed patients regains sensitivity after >1 year, and interestingly, that in some daratumumab‐refractory patients, isatuximab led to slightly better “ex vivo” results.^11^ Another “In vitro” studies performed in daratumumab‐refractory patients have demonstrated that CD38 mRNA is not downregulated or mutated in MM cells that maintain their surface expression of CD38 and for this reason CD38 remains a viable target in these patients.^12^ The same investigations have revealed that effector immune cells, such as CD8+ T cells and natural killer (NK) cells from nonresponding patients exhibit impaired functions (reduction or absence of MM cells killing capacity in an “in vitro” flow cytometry assay).^12^ Different strategies to overcome daratumumab‐resistance have been suggested, being one of them the use of other anti‐CD38 mAb with a different mechanism of action such as isatuximab.^13^, ^14^, ^15^ Isatuximab mediates a direct cytotoxicity against MM cells, in addition to the canonical Fc‐dependent mechanisms of action, it induces a CD38‐dependent depletion of MM cells via homotypic aggregation‐associated cell death by actin cytoskeleton polymerization, caspase‐dependent apoptosis, and lysosomal cell death; it also induces an allosteric modulation of CD38 that results in a higher inhibition of its ecto‐enzymatic activity, and in clinical trials, isatuximab has demonstrated a great antitumoral activity alone or in combination with IMiDs or PIs.^13^ PIs show substantial efficacy in combination with isatuximab, likely due to their multiple effects on MM cells and the tumor microenvironment.^15^

In an effort to improve the response before performing an autologous hematopoietic stem cell transplantation, we discussed the second therapy line financed in Spain among the following: pomalidomide‐bortezomib‐dexamethasone (PVD), carfilzomib‐dexamethasone (KD), isatuximab‐pomalidomide‐dexamethasone (ISA‐PomaDexa), or isatuximab‐carfilzomib‐dexamethasone (ISA‐KD). The inclusion of the patient in a clinical trial (e.g., anti‐BCMA CAR‐T, a CD3xBCMA bispecific monoclonal antibody, or a venetoclax‐based combination) was also considered.^16^, ^17^, ^18^, ^19^

Finally, we decided to administer the triplet combination of ISA‐KD, with a once‐weekly carfilzomib dose of 70 mg/m^2^ on days 1, 8, and 15 according to the A.R.R.O.W. scheme every 4 weeks.^6^ Surprisingly, a rapid and deep response was observed after the first ISA‐KD cycle that preceded the achievement of a VGPR after four ISA‐KD cycles (see biochemical evolution in Figure 1).

Recently Taku Kikuchi et al, have reported findings from the largest real‐world clinical analysis of 39 RRMM who were treated with isatuximab combinations, predominantly ISA‐Poma‐Dexa subsequent to daratumumab‐containing therapies.^5^ In this retrospective single center study that included RRMM with a median of 4 prior therapy lines, the worst outcomes were observed in daratumumab‐refractory or triple class‐refractory patients as well as those with high lactate dehydrogenase (LDH) levels or those who received isatuximab in the 3 months after daratumumab.^5^

Although we cannot strictly consider our patient to meet the criteria of daratumumab‐refractory or triple‐class‐refractory MM (because at least a minimal response was achieved after four cycles of Dara‐VRD), an early use of an alternative anti‐CD38 containing combination (ISA‐KD) has produced a rapid and deep response. This case illustrates that in non‐heavily pretreated NDMM, a washout period may not be strictly necessary. For NDMM patients with poor responses following the quadruplet Dara‐VRD, the ISA‐KD combination might be an excellent rescue therapy. This approach facilitated the achievement of an optimal response prior to the planned ASCT.

## AUTHOR CONTRIBUTIONS

**Juan Jose Gil‐Fernandez:** Formal analysis; investigation; methodology; validation; writing – original draft; writing – review and editing. **Patricia Garcia Ramirez:** Validation; writing – review and editing. **Marta Callejas Charavia:** Validation; writing – review and editing.

## FUNDING INFORMATION

No funding received.

## CONFLICT OF INTEREST STATEMENT

GFJJ: Honoraria from Janssen, Bristol Myers Squibb, GlaxoSmithKline, Sanofi, and Amgen. GRP and CChM: no relevant financial relationships to disclose.

## ETHICAL APPROVAL

This study has been performed according to the declaration of Helsinki.

## CONSENT

Written informed consent was obtained from the patient to publish this report in accordance with the journal's patient consent policy.

## Data Availability

Although this is a single clinical observation, patient's data are available for further purposes.
